# Epidemiological analysis of 2,368 pesticide poisoning patients in Quzhou City, China

**DOI:** 10.3389/fpubh.2025.1587271

**Published:** 2025-05-30

**Authors:** Xiawen Zheng, Ting Zheng, Lushan Wang, Shiling Jiao, Xianchen Jiang, Shiming Lai, Bingdong Zhan

**Affiliations:** ^1^Occupational Health Department, Quzhou Center for Disease Control and Prevention, Quzhou, China; ^2^Department of Chronic Disease Prevention and Control, Quzhou Center for Disease Control and Prevention, Quzhou, China

**Keywords:** pesticide, poisoning, fatality, herbicides, non-occupational

## Abstract

**Introduction:**

Pesticides, which are chemical compounds, are mainly utilized to eradicate pests such as insects, rodents, fungi, and unwanted plants (weeds). However, improper application or storage of pesticides can lead to poisoning incidents. In China, many patients tend to abandon treatment and return home when their condition severely deteriorates. This behavior makes it challenging for medical institutions to precisely track the patients’ subsequent conditions, resulting in the reported number of deaths in the system being lower than the actual figure.

**Methods:**

This research obtained case data on pesticide poisoning in Quzhou city from 2015 to 2022 (2,368 confirmed cases) from ODSRS, using patient ID card numbers on the report cards to match cause-of-death data in the Zhejiang Chronic Disease Monitoring Information Management System. Excel 2013 was used for database establishment, Graph Prism 9.5.0 for statistical analysis and graphing, and ArcMAP10.2 for creating regional distribution maps. The chi-square test compared categorical variable groups; binary logistic regression explored factors influencing pesticide poisoning mortality.

**Results:**

From 2015 to 2022, a total of 2,368 pesticide poisoning cases were documented in Quzhou City, exhibiting a downward trend. Among them, 280 patients died, with a case-fatality rate of 11.82%. The fatality rate was higher in males (13.35%) compared to females (10.03%), and it increased with age. Insecticides were implicated in 66.05% of the poisoning cases, followed by herbicides (20.82%) and rodenticides (9.71%). Notably, herbicides had the highest fatality rate at 15.21%. Non-occupational poisoning accounted for 91.01% of the cases, with suicidal poisoning constituting 65.57% and having a fatality rate of 15.07%. Statistically significant differences were observed in the distributions of fatalities across different genders, age groups, pesticide types, and causes of poisoning (*p* < 0.05). Pesticide poisoning was reported in all six counties of Quzhou City, with Kaihua County having the highest incidence, mortality, and case-fatality rate.

**Conclusion:**

This study indicates that the actual fatality rate of pesticide poisoning patients is substantially higher than the reported rate. Additionally, being male, over 40 years old, and having non-occupational exposure to herbicides were associated with higher death odds ratios.

## 1 Introduction

Pesticides are chemical compounds that are most commonly used to kill pests, including insects, rodents, fungi and unwanted plants (weeds). Currently, given the high-productivity requirements in agriculture, pesticides have become an indispensable choice. Over 1,000 different pesticides are used worldwide (1). Improper use or storage of pesticides can lead to poisoning. Poisoning of non-targets in relation to improper use of pesticides (2). Pollinators are a relatively common non-target of acute poisoning from pesticide use, especially if they are used improperly (3). Human pesticide poisoning can be categorized into two major types according to the cause: occupational and non-occupational (4). Occupational poisoning can occur when, during agricultural production, there is a lack of protective equipment, resulting in respiratory inhalation or skin contact with pesticides. Non-occupational poisoning, which occurs due to unintentional or intentional skin contact, oral ingestion, or respiratory tract inhalation of pesticides in daily life, can be further divided into accidental and suicidal types. According to one study, the proportion of pesticide-related suicides in rural areas is much higher than that in urban areas (5).

China is a major agricultural country, with an annual pesticide consumption of approximately 1.4 million tons (6). The Occupational Disease Surveillance and Reporting Systems (ODSRS) (7) was launched in 2006 to collect information on occupational hazards and poisoning (8). Based on systematic data, a large number of studies on pesticide poisoning have been published (9, 10). However, many Chinese patients may choose to abandon treatment and return home when their condition deteriorates severely. This situation makes it difficult for medical institutions to accurately understand the patient’s subsequent status. As a result, the number of reported deaths in the system is lower than the actual number.

The Zhejiang Chronic Disease Monitoring Information Management System was established by the Zhejiang Center for Disease Control and Prevention (Zhejiang CDC) in 2002. Initially, the system covered only 34.8% of the Zhejiang Province population. Eventually, by 2010, it achieved 100% population coverage, with Quzhou City being included in 2009 (11). The reporting rate of mortality monitoring data from 2016 to 2018 was 99% (12). Since 2015, the ID number has been made a mandatory entry in the ODSRS. Therefore, we integrated the ODSRS with the Zhejiang Chronic Disease Monitoring Information Management System to obtain accurate fatality data for pesticide-poisoning cases from 2015 to 2022. Based on these accurate data, we explored the factors influencing the fatality rate of pesticide poisoning cases in Quzhou city, China.

## 2 Materials and methods

### 2.1 Study areas

The city of Quzhou is the source of the Qiantang River, covering 8,844 km^2^ in western Zhejiang Province, China, where the gross domestic product (GDP) is expected to reach 200.3 billion yuan in 2022. According to the National Bureau of Statistics, the resident population of Quzhou city in 2022 is 2.29 million, which includes 0.95 million rural residents. The urbanization rate of Quzhou City in 2022 was 59.3%, far below the average level of 73.4% in Zhejiang Province (13).

### 2.2 Data sources

Case data on pesticide poisoning in Quzhou city from 2015 to 2022 were obtained from the ODSRS, and 2,368 cases were confirmed in this study. All cases underwent diagnosis by specialists at different hierarchical levels within the hospital, following the corresponding national diagnostic guidelines. The pesticide poisoning report card included the patient’s ID card number and poisoning date, and we used the ID card number to match the cause-of-death surveillance data in the Zhejiang Chronic Disease Monitoring Information Management System to obtain the accurate date and cause of death.

For the purpose of this study, patients were classified as dying from pesticide poisoning if they met the following criteria: (1) they succumbed within 30 days of poisoning; (2) the underlying cause of death was determined to be associated with pesticide poisoning, as evidenced by the assignment to relevant ICD - 10 codes (T60, X48, X49, X68, and Y18). Exclusion criteria: (1) Repeatedly reported cases. Duplicates were checked by using ID card numbers. (2) Non - pesticide poisoning cases (such as drug poisoning, alcohol poisoning, etc.). To ensure the comprehensiveness and reliability of the report data, all pesticide poisoning report cards and death cards uploaded to the system were reviewed by physicians at the CDC at the county/district, city, provincial and national levels. The calculation of the incidence rate and mortality was based on the average resident population of Quzhou from 2015 to 2022.

### 2.3 Case definition

The pesticide poisoning report cards included basic information, such as age, sex, type of pesticide and cause of poisoning. The age was classified as follows: under 20 years, 20–40, 41–60 and over 60 years. The causes of pesticide poisoning can be divided into two categories: occupational and non-occupational. Non-occupational cases can be divided into accidental and suicide. Pesticides mainly included insecticides, herbicides, rodenticides and others (including bactericides, biochemical pesticides and unknown agents).

### 2.4 Data analysis

Excel 2013 was used to establish a case database, Graph Prism 9.5.0 was used for statistical analysis and graph drawing, and regional distribution maps were produced using ArcMAP10.2. The chi-square test was used to compare the differences between groups of categorical variables. Binary logistic regression analysis was conducted to explore the factors affecting the fatality of pesticide poisoning. The dependent variable was pesticide deaths (vs no deaths). Logistic regression models were presented as odds ratios (OR) and 95% confidence intervals (13). A two-tailed *p* < 0.05 was considered to indicate statistical significance.

## 3 Results

### 3.1 Basic information

From 2015 to 2022, there were 2,368 cases of pesticide poisoning in Quzhou city, Zhejiang Province, China. A total of 280 patients died, for a case fatality of 11.82%. Among the patients, 1,281 were male and 1,087 were female; the fatality was significantly greater in males (13.35%) than in females (10.03%) (*p* = 0.013). The age range of the patients was 0–94 years, with an average age of 52.71 years. The fatality increased with age, from 1.4% for those <20 years old to 19.29% for those >60 years old. Occupational pesticide poisoning occurred in 213 patients, and 2,155 were considered non-occupational. Among those in the non-occupational pesticide poisoning group, 213 patients died from suicide, with the highest fatality of 15.07%. Moreover, 742 patients were accidentally poisoned, for a fatality of 8.49%. The differences in the distribution of fatalities according to sex, age group and cause of poisoning (*p* < 0.05) were statistically significant, as shown in Table 1.

**Table 1:** Basic demographic characteristics of pesticide poisoning cases in Quzhou from 2015 to 2022.

Basic information		Case number	Death number	Fatality (%)	*χ* ^2^	*p*
Sex	Male	1,281	171	13.35	6.222	0.013
	Female	1,087	109	10.03		
Age (years)	<20	214	3	1.40	98.768	<0.001
	20 ~ 40	431	23	5.34		
	41 ~ 60	743	65	8.75		
	>60	980	189	19.29		
Cause of Poisoning	Occupational poisoning	213	4	1.88	42.435	<0.001
	Non-Occupational (accident)	742	63	8.49		
	Non-Occupational (suicide)	1,413	213	15.07		
Total		2,368	280	11.82		

**Figure 1:**
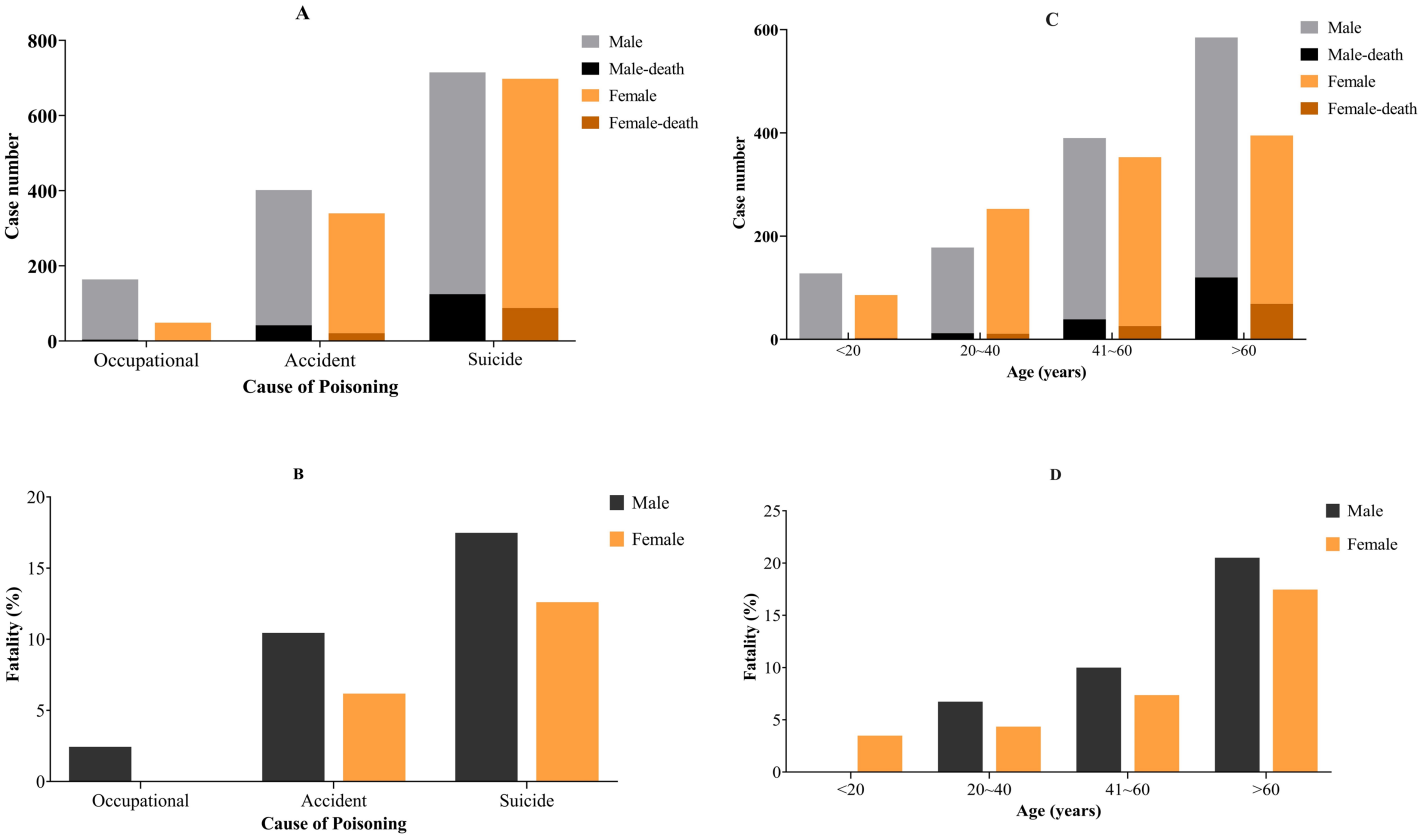
Fatality of pesticide poisoning among different genders in Quzhou city. **(A,B)** Different causes of poisoning; **(C,D)** different age group. The incidence and case fatality rate of pesticide - related suicide are the highest. Both the number of pesticides poisoning cases and the case fatality increase with age.

Figure 1 shows the fatality of pesticide poisoning among the different sexes. The fatality of males in all poisoning cause groups were greater than that of females (Figures 1A,B). In addition, there were greater fatality in males than females in all age groups except for those <20 years (Figure 1C).

### 3.2 Types of pesticides causing poisoning

**Figure 2:**
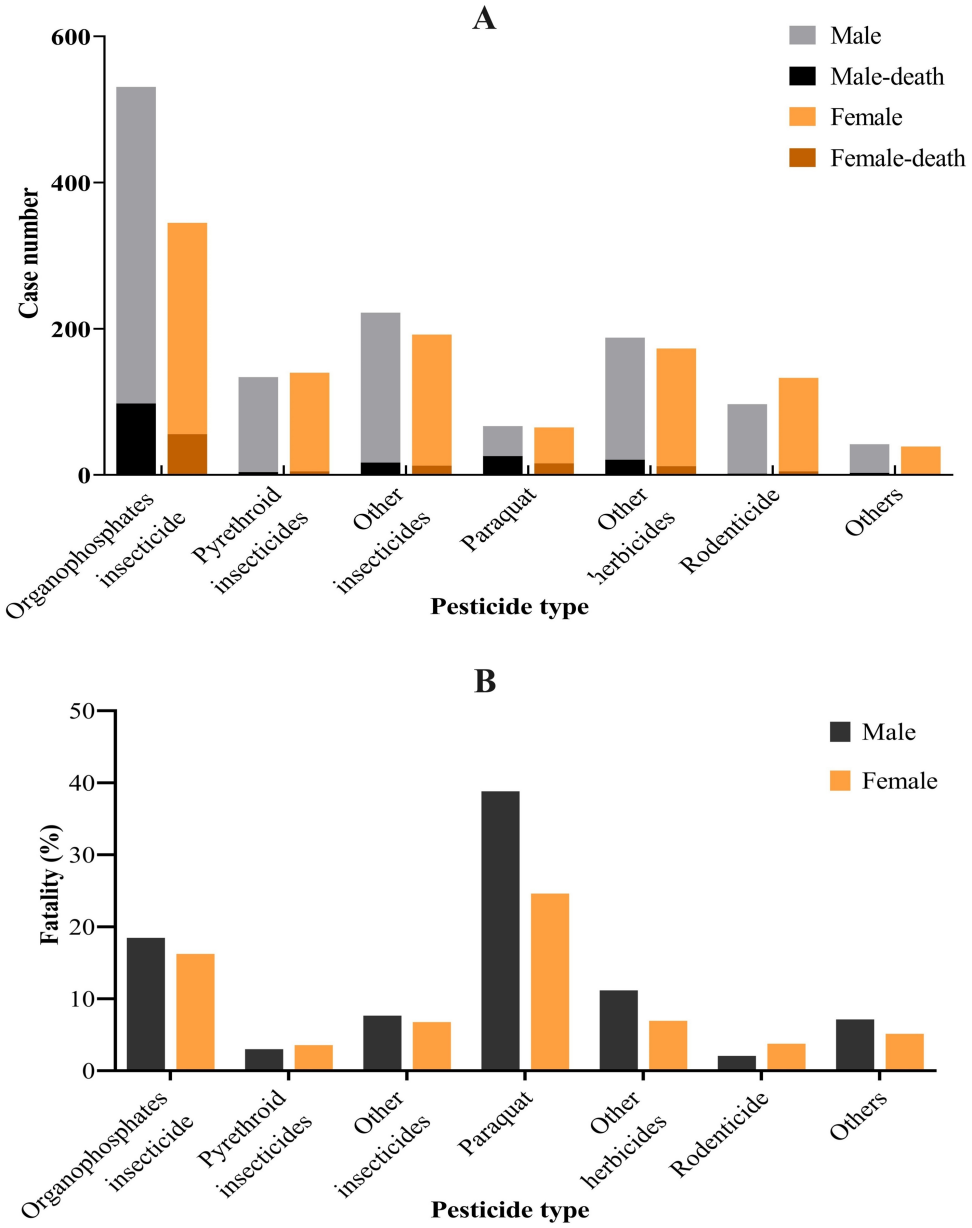
Fatality of pesticide poisoning among different pesticidetypes in Quzhou city. **(A)** Case number; **(B)** fatality. The number of people poisoned by organophosphate insecticides is the highest, and paraquat has the highest case fatality.

In our study, pesticide poisoning was mostly caused by insecticides, which accounted for 1,564 cases (66.05% of all the cases), followed by herbicides (493, 20.82%) and rodenticides (230, 9.71%). Paraquat had the highest fatality, followed by organophosphates insecticide, with fatality of 31.82 and 17.58%, respectively. The fatality of rodenticides was the lowest, at 3.04%, as shown in Table 2 and Figure 2.

**Table 2:** Pesticide types involved in pesticide poisoning in Quzhou from 2015 to 2022.

Type	Case number	Death number	Fatality (%)	*χ* ^2^	*p*
Insecticide	1,564	193	12.34	127.913	<0.001
Organophosphates insecticide	876	154	17.58		
Pyrethroid insecticides	274	9	3.28		
Other insecticides	414	30	7.25		
Herbicides	493	75	15.21		
Paraquat	132	42	31.82		
Other herbicides	361	33	9.14		
Rodenticide	230	7	3.04		
Others	81	5	6.17		
Total	2,368	280	11.82		

### 3.3 Trend of pesticide poisoning

**Figure 3:**
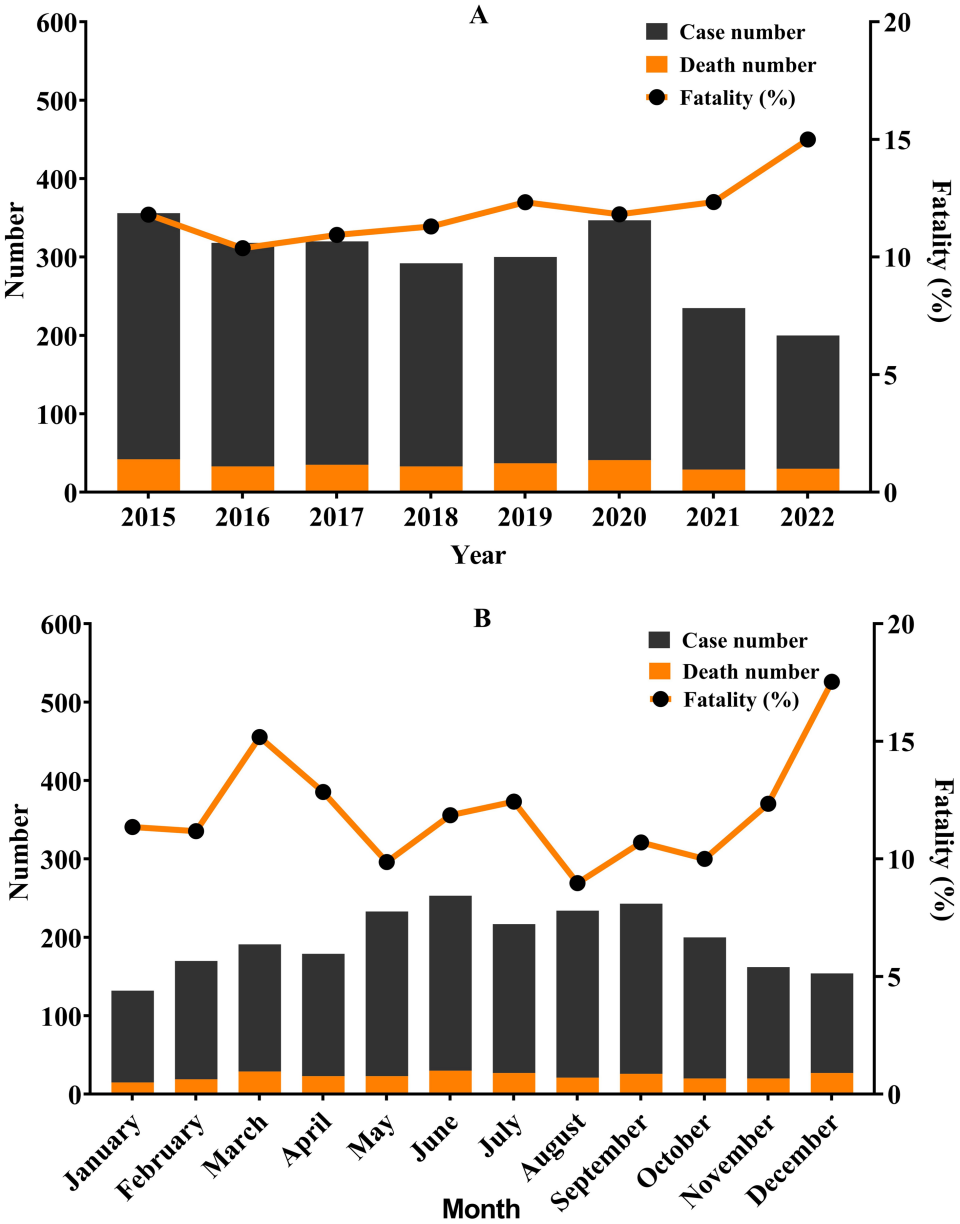
Pesticide poisoning cases and fatalities in Quzhou from 2015 to 2022 by month and year. **(A)** Year; **(B)** month. Fatality appears to rise despite falling case numbers.

The number of cases per year showed a slow downward trend, from 356 in 2015 to 200 in 2022. However, the fatality increased from 11.80% in 2015 to 15.00% in 2022, without a significant difference (*χ^2^* = 3.026, *p* = 0.883), as shown in Figure 3A. The highest number of reported cases occurred in summer, from June to September. 10.68 and 10.26% of all cases in June and September, respectively. The highest fatality was observed in December (17.53%), as shown in Figure 3B. However, there was no statistically significant difference in monthly fatality (*χ^2^* = 10.886, *p* = 0.453).

### 3.4 Regional distribution of pesticide poisoning

**Figure 4:**
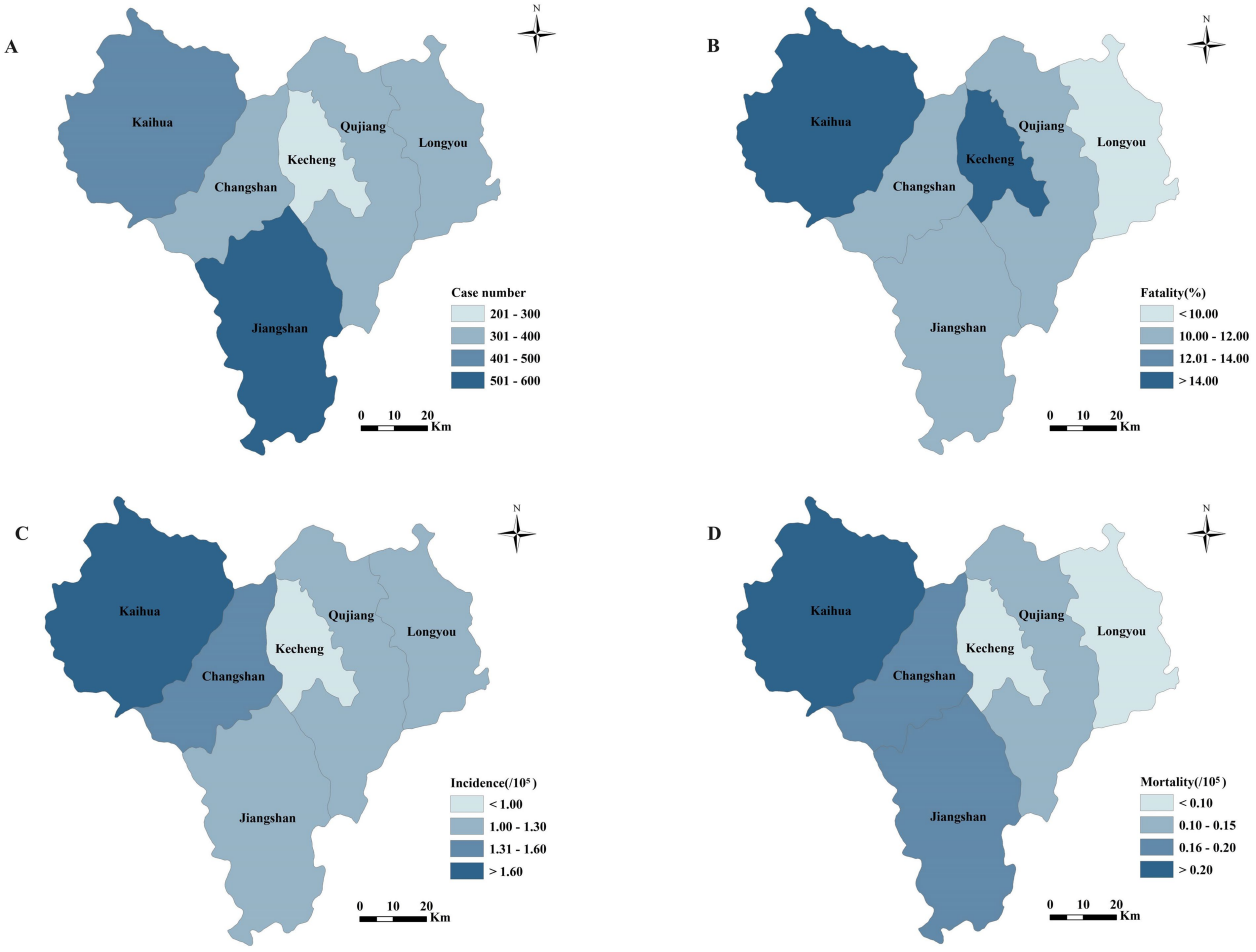
Regional distribution of pesticide poisoning cases reported in Quzhou city from 2015 to 2022. **(A)** Case number; **(B)** fatality; **(C)** incidence; **(D)** mortality. Map created using ArcMAP10.2 (https://downloads.esri.com/archydro/archydro/Setup/ArcMap/10.2/). The incidence rate, mortality and case fatality of Kaihua County ranked first in Quzhou city.

Figure 4 shows the number of cases and fatality of pesticide poisoning in various regions of Quzhou city. The two countries with the most reported cases of pesticide poisoning from 2015 to 2022 were Jiangshan (585 cases, 24.70%) and Kaihua (413 cases, 17.44%). In addition, Kecheng had the lowest number of cases (224 cases, 9.46%), incidence rate and mortality but the highest fatality (15.18%). It was obvious that the incidence rate, mortality and case fatality of Kaihua County ranked first in Quzhou city. There was a statistically significant difference of fatality among different regions (*p* = 0.034).

**Figure 5:**
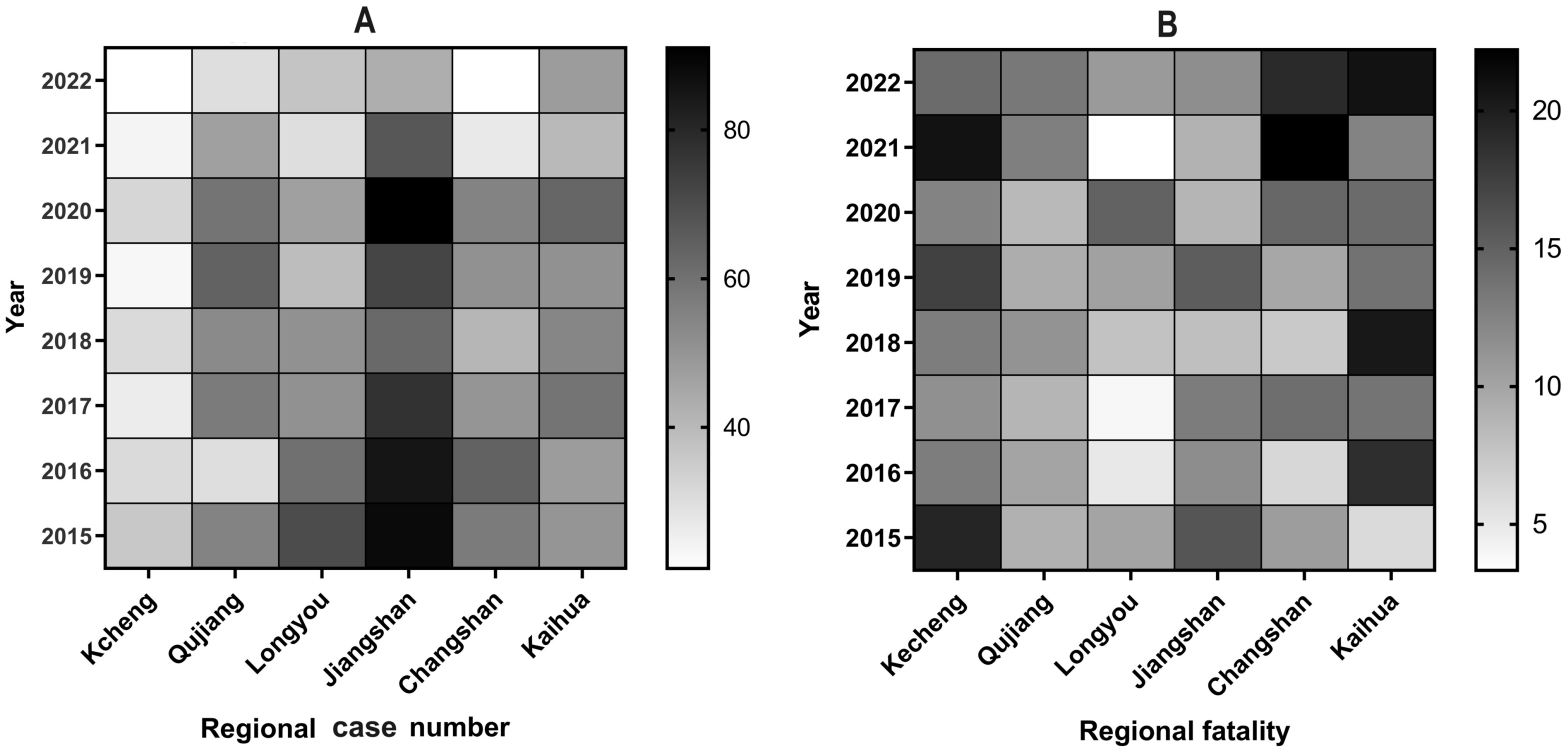
**(A)** and **(B)** Regional distributions of reported cases and fatality due to pesticide poisoning, respectively, in Quzhou city from 2015 to 2022. Jiangshan had the highest number of cases per year, while the fatality of Kaihua and Kecheng were greater than those of other counties.

Figure 5 shows the annual incidence and fatality in various regions. Jiangshan had the highest number of cases per year, while the fatality of Kaihua and Kecheng were greater than those of other counties.

### 3.5 Influence factor of pesticide poisoning

Based on the available data, the potential risk factors influencing the case fatality of pesticide poisoning patients were sex, age, type of pesticide and cause of poisoning. Moreover, male sex, age >40 years, and non-occupational exposure to herbicides had higher odds ratios of death, as shown in Table 3.

**Table 3:** Binary logistic regression analysis.

Influence factor		OR	95%CI	*p*
Sex	Female			1.000
	Male	1.348	1.032–1.761	0.029
Age (year)	<20			1.000
	20–40	2.726	0.797–9.327	0.110
	41–60	5.050	1.550–16.456	0.007
	>60	13.824	4.323–44.200	<0.001
Pesticides type	Other			1.000
	Insecticides	1.948	0.762–4.979	0.164
	Herbicides	3.056	1.168–7.993	0.023
	Rodenticide	0.475	0.143–1.573	0.223
Cause of poisoning	Occupational			1.000
	Non-Occupational(accident)	6.903	2.458–19.390	<0.001
	Non-Occupational(suicide)	12.146	4.433–33.281	<0.001

## 4 Discussion

This article included a total of 2,368 cases of pesticide poisoning from 2015 to 2022. Among them, 280 patients died, resulting in a case-fatality rate of 11.82%, which was much greater than that in other regions of China (14). For example, the fatality was 5.5% in Jiangsu Province from 2007 to 2016 (6), and 7.03% in Zhejiang Province from 2006 to 2010 (8). One key difference of this study was the integration of cause-of-death surveillance data, which provided more accurate and comprehensive death outcomes. This might be the reason for the relatively high reported fatality. The combination of multiple data sources was crucial for improving the accuracy of fatality estimation in epidemiological research.

### 4.1 Epidemiological distribution of pesticide poisoning

The sex distribution indicated that the number of poisoned individuals and the mortality rate were higher for males than for females. This could be because males have more opportunities to use pesticides than females in agricultural production processes. The age distribution showed that both the number of cases and the fatality increased with age, especially for older adults older than 60 years. Older adults typically have poorer health conditions, lower therapeutic motivation, and a poorer prognosis, leading to a higher fatality rate.

The most common type of pesticide poisoning was insecticide poisoning, accounting for 66.05% of cases, followed by herbicide poisoning, accounting for 20.82%. Overall, the fatality of herbicides (15.21%) was higher than that of insecticides (12.34%). This result was consistent with other studies, which indicated that certain herbicides have higher lethality. Among them, paraquat had a particularly significant fatality of 31.82%. This fact highlights the necessity for stricter regulation of the use of paraquat. Paraquat is one of the most widely used herbicides for the control of weeds in many agricultural and non-agricultural settings. Its broad-spectrum weed-killing ability and relatively low cost made it popular among farmers for a long time. However, the extremely high toxicity of paraquat to humans, especially the lack of effective antidotes, has led to numerous tragic cases of poisoning. Even small amounts of ingestion can cause irreversible damage to multiple organs, often resulting in a high fatality rate. The Ministry of Agriculture and Rural Affairs of the People’s Republic of China has issued a notice to stop the sale and use of paraquat in China from July 1, 2016 (15). This decision was made after comprehensive consideration of its potential risks to public health and the environment. Since the implementation of this ban, the number of paraquat-related poisoning cases has decreased significantly. Nevertheless, there are still challenges in ensuring complete compliance. Some illegal channels may still be involved in the smuggling or illegal production and sale of paraquat. Therefore, continuous efforts are needed in terms of law enforcement to prevent the re-emergence of paraquat in the market.

From 2015 to 2022, 91.05% of the total cases were non-occupational pesticide poisoning. Among them, 65.57% were suicide-related poisonings. The fatality of suicide poisoning was 7 times higher than that of productive-related poisonings and 1.77 times greater than that of accidental poisoning. From 2006 to 2013, the most common method of suicide in China was pesticide poisoning, and the suicide rates increased exponentially with age (14). A retrospective observational study in South Korea showed that intentional poisoning cases had a higher fatality rate and more major adverse events than unintentional poisoning cases (16). Compared to occupational poisoning and accidental poisoning, suicide often involves the ingestion of a larger dosage of pesticides. This may be the main reason for the higher fatality rate.

There were significant regional differences in the incidence and mortality rates of pesticide poisoning in Quzhou City. Jiangshan had the largest number of cases, with 583. Kaihua and Kecheng had the highest fatality, both over 15%. The incidence rate, mortality rate, and case-fatality rate in Kaihua County ranked first in Quzhou City. This may be related to the economic foundation and agricultural development level of the regions. Studies have demonstrated a greater suicide risk among farmers than among the general adult population (17). Kaihua is a rural county located in the northwestern part of Quzhou City. The city has poor economic conditions and lower medical treatment levels, making it difficult to transport patients to the hospital over long distances. Kecheng District is the main urban area of Quzhou City and is responsible for the treatment of severe cases surrounding the countryside; this may lead to a higher fatality.

From 2015 to 2022, the overall number of pesticide-poisoning cases in Quzhou City showed a slight downward trend. These findings were consistent with the conclusions of a similar study in Jiangsu Province (6). According to a report from the Ministry of Agriculture and Rural Affairs of the People’s Republic of China, the amount of pesticide applied in China has been decreasing annually. According to monitoring statistics, the pesticide use in 2019 was 262,900 tons, a decrease of 37,000 tons compared to 2015 (18). The reduction in pesticide use is beneficial to reducing the pesticide-related suicide rate (13). Despite the positive impact of these regulatory measures, it is worth noting that pesticide suicide remains a significant public health issue globally. In fact, intentional use of pesticides for suicide accounts for one-third of all suicides worldwide (19). A systematic review estimated that approximately 44% of farmers are poisoned by pesticides every year (20). In Pakistan, 47% of poisonings are caused by pesticides (21). Therefore, efforts to reduce pesticide-related deaths should continue to focus on strengthening regulation and public health interventions, especially on addressing potential mental health and socio-economic factors that lead to suicide.

### 4.2 Preventive measures and intervention strategies

To reduce the incidence of pesticide poisoning, it is recommended to take a series of preventive measures. First, increasing the use of personal protective equipment when handling for pesticides can reduce the incidence of occupational pesticide poisoning among farmers (22). Second, better management of pesticides can reduce cases of non-occupational pesticide poisoning caused by accidents. This includes new closed-system packaging designed to make it impossible to transfer or remove the pesticide except directly into the proper application equipment; special training for certified applicators who use pesticide to emphasize that the chemical must not be transferred to or stored in improper containers; and changes to the pesticide label and warning materials to highlight the toxicity and risks associated with pesticide (23). However, a study in rural Asia showed that improving pesticide storage methods in households does not effectively reduce pesticide poisoning (24). Therefore, for pesticide suicides with the highest fatality, multiple methods should be adopted. Restricting the use of lethal means will reduce suicide (25). If highly toxic pesticides were replaced, 150,000 pesticide suicides could be avoided worldwide each year (26). Many countries have implemented strategies to reduce cases of intentional poisoning, such as deregistering lethal pesticides, banning their sale on the market, and restricting production (27–29). These measures have been effective in reducing the number of pesticide-related suicides globally, but the incidence of ingestion and deaths remains high. Research has shown that pesticide-related deaths are mostly impulsive, and that promoting the use of low-toxicity pesticides can increase patient survival rates. Improving the provision of medical care in resource-poor hospitals can improve the cure rate. To reduce the incidence of repeat suicide, appropriate psychological intervention and physical therapy should be carried out. Different interventions should be implemented for different patient situations (30).

### 4.3 Study limitations and future research directions

This study provides valuable insights into the fatality and risk factors associated with pesticide poisoning in Quzhou City. However, it does have several limitations. First, the study’s heavy reliance on data from the Zhejiang Provincial Chronic Disease Surveillance Information Management System has a potential drawback. This system comprehensively records hospital-based cases but fails to account for pesticide poisoned individuals who did not seek treatment at formal healthcare facilities. As a result, the actual incidence of pesticide poisoning is likely underestimated, as cases that occurred outside the hospital setting are excluded. Second, the geographical scope of this research is restricted to Quzhou City. Although Quzhou represents a particular geographical and demographic profile, it cannot fully mirror the diverse situations in other regions. The narrow regional coverage may limit the generalizability of the study findings, as different areas may have distinct agricultural practices, pesticide usage patterns, and environmental factors that could influence pesticide-related health outcomes. Third, the research mainly concentrates on the basic information and short-term outcomes of pesticide poisoning cases, overlooking the long-term health implications. Pesticide exposure can potentially trigger chronic health issues, including neurological disorders, endocrine disruption, and an elevated risk of certain cancers. Understanding these long-term effects is crucial for a comprehensive assessment of the impact of pesticide poisoning on public health. In terms of the applicability of the results, due to the limited geographical scope and data source, they are more likely to be relevant to areas within Zhejiang Province that have similar agricultural activities and healthcare systems. However, with appropriate adjustments and further validations, some of the findings may offer valuable references for other regions with comparable pesticide-use patterns. For future research, it is essential to adopt more comprehensive data-collection methods. Integrating community-based monitoring approaches could significantly improve the case-capture rate. By including cases from the community level, researchers can obtain a more accurate and complete picture of pesticide-related health problems. Additionally, establishing long-term follow-up mechanisms for pesticide-poisoned patients is highly recommended. This would enable a better evaluation of the long-term health impacts, providing more in-depth knowledge for the prevention and management of pesticide-related health risks.

## 5 Conclusion

The study found that male, age over 40, and non-occupational exposure to herbicides were associated with higher fatality from pesticide poisoning. The overall fatality in Quzhou was high, which highlights the necessity of taking further preventive measures, especially reducing access to pesticides and strengthening mental health support to address self-poisoning cases. Additionally, long-term follow up should be conducted for pesticide poisoning patients to assess their long-term health impacts and provide deeper insights into the prevention and management of pesticide related health risks.

## Data Availability

The datasets presented in this article are not readily available because ODSRS and Zhejiang Chronic Disease Monitoring Information Management Systems are encrypted and closed to public access. The datasets used and/or analyzed during the current study are available from the corresponding author upon reasonable request. Requests to access the datasets should be directed to qzcdctg@163.com.
